# Effect of speech-stimulus degradation on phoneme-related potential

**DOI:** 10.1371/journal.pone.0287584

**Published:** 2023-06-23

**Authors:** Min-Jae Jeon, Jihwan Woo

**Affiliations:** 1 Department of Electrical, Electronic and Computer Engineering, University of Ulsan, Ulsan, Republic of Korea; 2 Department of Biomedical Engineering, University of Ulsan, Ulsan, Republic of Korea; Hannover Medical School: Medizinische Hochschule Hannover, GERMANY

## Abstract

Auditory evoked potential (AEP) has been used to evaluate the degree of hearing and speech cognition. Because AEP generates a very small voltage relative to ambient noise, a repetitive presentation of a stimulus, such as a tone, word, or short sentence, should be employed to generate ensemble averages over trials. However, the stimulation of repetitive short words and sentences may present an unnatural situation to a subject. Phoneme-related potentials (PRPs), which are evoked-responses to typical phonemic stimuli, can be extracted from electroencephalography (EEG) data in response to a continuous storybook. In this study, we investigated the effects of spectrally degraded speech stimuli on PRPs. The EEG data in response to the spectrally degraded and natural storybooks were recorded from normal listeners, and the PRP components for 10 vowels and 12 consonants were extracted. The PRP responses to a vocoded (spectrally-degraded) storybook showed a statistically significant lower peak amplitude and were prolonged compared with those of a natural storybook. The findings in this study suggest that PRPs can be considered a potential tool to evaluate hearing and speech cognition as other AEPs. Moreover, PRPs can provide the details of phonological processing and phonemic awareness to understand poor speech intelligibility. Further investigation with the hearing impaired is required prior to clinical application.

## 1. Introduction

Speech perception, which is the ability to hear and process speech information, is widely evaluated using either behavioral or electrophysiological tests in clinical settings. Behavioral tests are simple and straightforward tasks that use sentences, words, and phonemes [1, 2]. Behavioral tests require the behavioral response of a subject, and hence, the outcomes may be affected by the subject’s condition [1–3]. Electroencephalography (EEG) is a popular method for measuring the electrical activity of the auditory nerve or brain in response to acoustic stimuli, which is typically known as auditory evoked potential (AEP). AEP has been reliably used in clinic settings and research.

The speech perception abilities of hearing aids and cochlear implant users have been systematically accessed by cortical event responses [4, 5]. Cortical AEP can be effectively used to understand speech discrimination and intelligibility of people for whom it is difficult to behaviorally test auditory function. However, because the measurement of AEP requires the use of electrodes placed on the scalp, which can result in a low signal-to-noise ratio, AEP requires repetitive presentation of a stimulus to obtain an ensemble average. An approach that uses repetitive stimuli may present an uncomfortable and unnatural situation for a subject [3, 6, 7]. Recently, continuous sentence stimuli, instead of a word or short-duration tone, have been used to access speech processing because they provide a more ecological measure of speech perception. The potential techniques, cortical tracking of speech envelope, temporal response function, and decoding speech from EEG can allow access to higher-level speech processing [3, 8, 9]. However, the AEP has a limitation in understanding phonemic processing at the cortical level and phonemic awareness in detail, which may be important for reading and spelling. It is also important to access the improvement of phonemic processing for users of hearing aids and cochlear implants to emphasize the benefit of auditory prosthesis [10, 11].

Recently, it has been investigated that the phoneme-related potential (PRP) can be obtained from time-locked responses to phoneme instances from a continuous storybook rather than repetitive stimulations of words and short sentences [12]. The findings of the above study provide evidence that phonetic information propagates along the auditory pathway and is subsequently encoded in the brain. The PRP showed morphological similarity to the P1-N1-P2 complex of auditory middle latency response. However, as the PRP in hearing-impaired and in response to degraded speech has not been investigated yet, it cannot be considered a possible tool to evaluate hearing and phonemic awareness. Each peak component of AEP is related to neural information processing, and an increase in the amplitude and decrease in the latency of each component generally denotes more informative processing in the human brain [13]. Therefore, we hypothesize that PRP, similar to other types of AEPs, can cause changes in peak amplitude and latency dependent on phonological processing. In this study, we investigated the effect of spectrally degraded speech on the amplitude and latency of PRP. A continuous storybook evoked EEG data from normal listeners were recorded to extract the PRP components for 10 vowels and 12 consonants. The results showed that the PRP responses to a vocoded (spectrally-degraded) storybook resulted in a statistically significant lower peak amplitude and were prolonged compared with those of a natural storybook. Finally, this study provided evidence on the feasibility of using PRP as an objective test for speech perception and as a useful tool for understanding phonemic awareness.

## 2. Methods

### 2.1. Participants

Twenty subjects (21.4±1.7 years old, 10 males and 10 females) participated in this study. All participants had no speech or hearing impairment and were native Korean speakers. The experimental procedures were reviewed and approved by the Institutional Review Board of the University of Ulsan and all participants signed an informed consent.

### 2.2. Natural and vocoded continuous storybook stimuli

**Fig 1:**
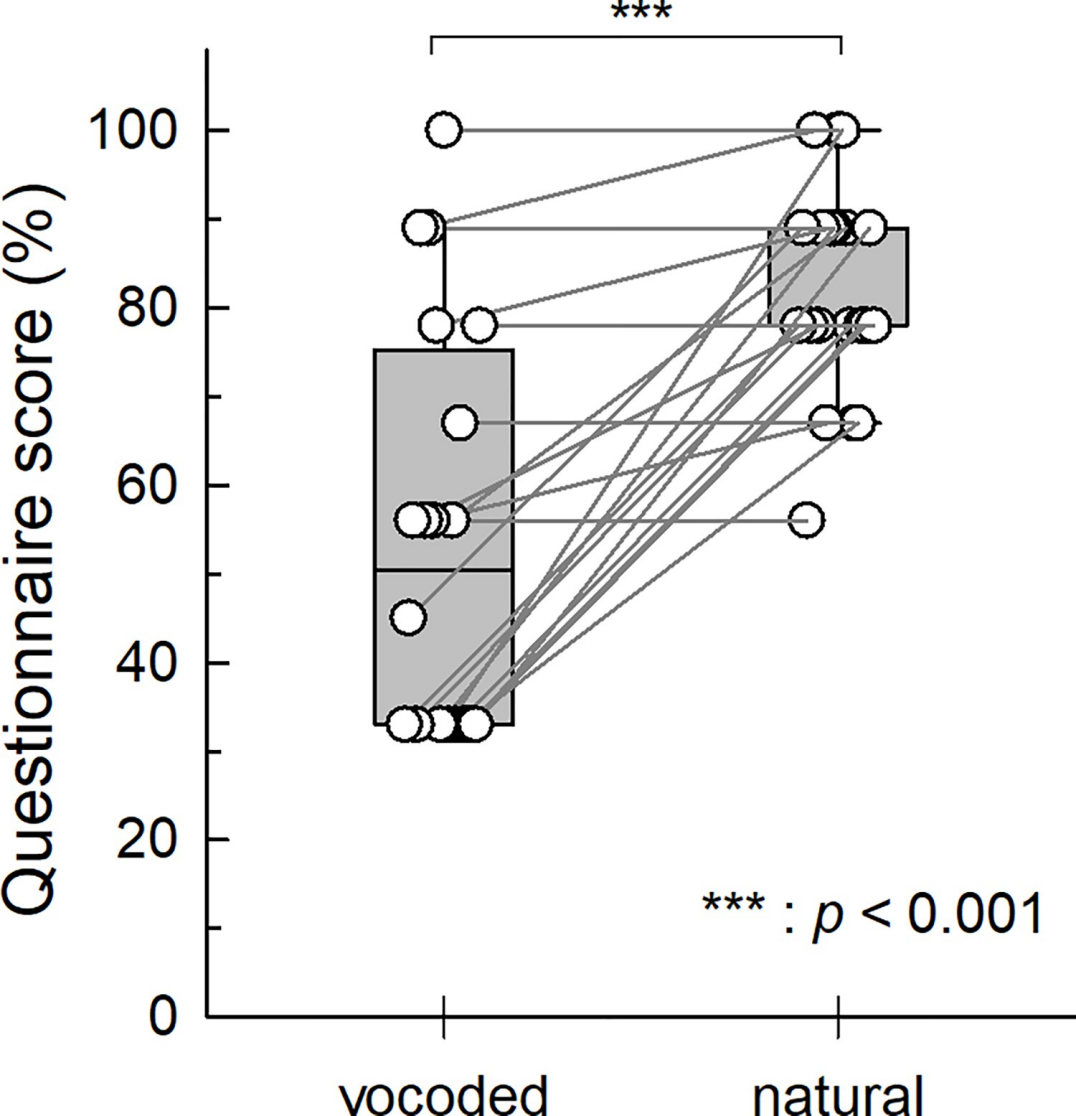
Comparison of questionnaire scores between the vocoded and natural cases using the Wilcoxon signed rank test (*n* = 20).

A female speaker recorded the stimulus storybook. The storybook consisted of noise-vocoded speech with degraded spectral detail and natural speech to provide different intelligible speech conditions [14]. The natural storybook was spectrally degraded using a noise vocoder. It was filtered using eight bandpass filters whose cutoff frequencies were logarithmically spaced between 200 Hz and 5000 Hz. After modulation with white Gaussian noise, the filtered data were synthesized for the vocoded storybook. The noise-vocoded speech story consisted of 398 sentences and the natural speech story consisted of 458 sentences. The duration of each storybook was 30 min. The storybook was presented at a comfortable hearing level of 60–70 dBA using a loudspeaker placed 1 m from the participant in a soundproof room. The participants were asked to listen attentively to the story and watch the cross on the monitor. The experiment consisted of six sessions (10 min per session) with a 5-min rest between sessions. A questionnaire was provided during each rest to confirm the attentiveness of the participants. The questionnaire consisted of nine questions about the story, and each question was designed to evaluate whether the story was comprehensible. The scores of each subject are detailed in Fig 1. The score for the vocoded story (mean: 53.3) is typically lower compared that of the natural story (mean: 81.5) (*p* < 0.001, Wilcoxon Signed Rank Test), indicating that there is a difference in understanding between the two cases.

### 2.3. Electroencephalography

Brain activity in response to storybook stimulation was measured using a 64-channel EEG system at a rate of 2048 Hz (Biosemi Co., Netherlands). The EEG data were preprocessed using common referencing and 2–57 Hz bandpass filtering for baseline correction. The data were resampled to 256 Hz to enhance computational efficiency. Eye blink-related noise was removed using independent component analysis [15].

### 2.4. Phoneme-related potential

**Fig 2:**
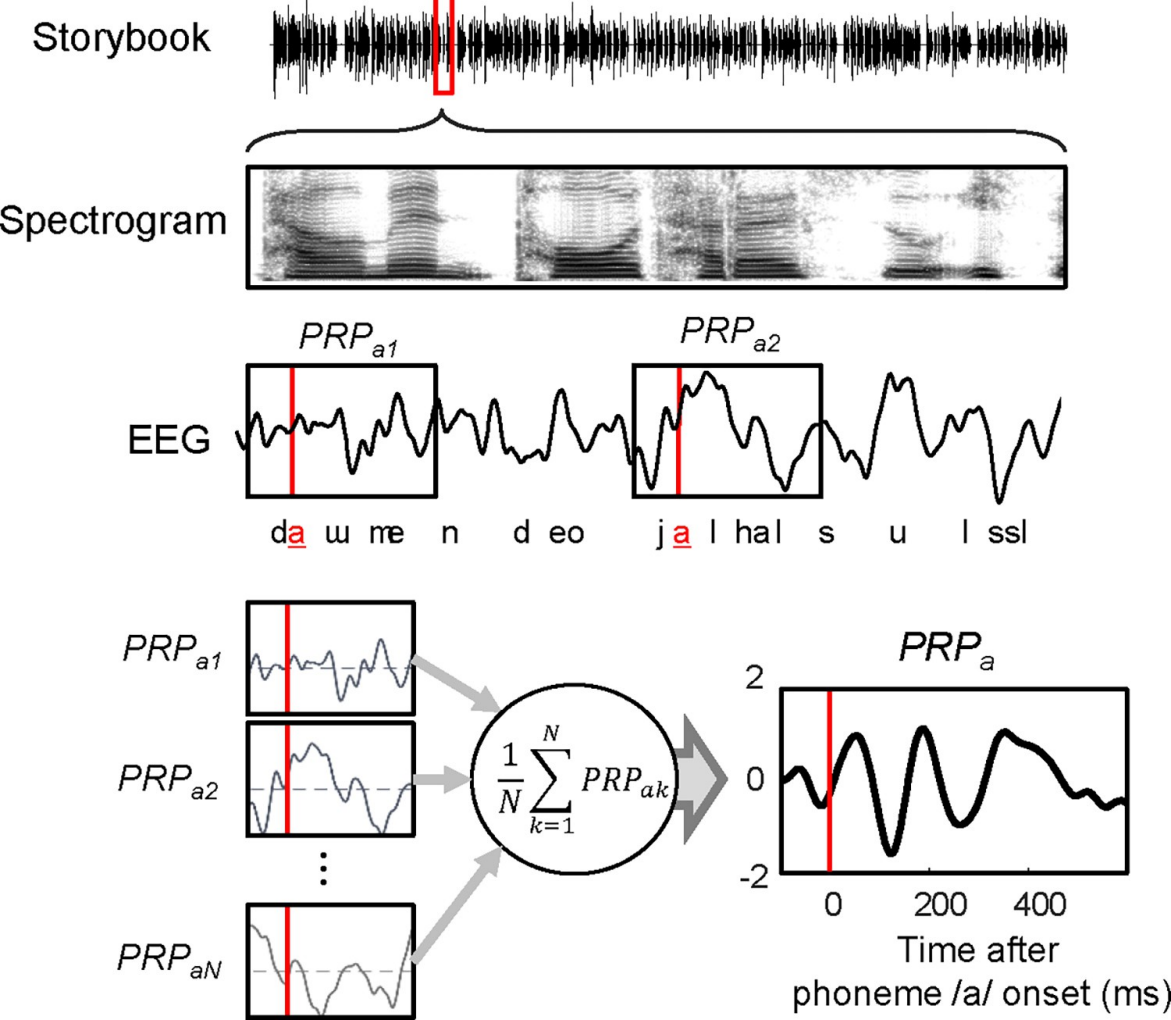
Example of the computation of the phoneme related potential (PRP) in response to the typical phoneme of ‘ㅏ /a/’. The stimulus spectrograms of part of the storybook are shown in the two upper panels, and the following panel shows a plot of details of electroencephalography (EEG) data in response to storybook stimuli. The EEG data were segmented based on the onset time of phoneme ‘ㅏ /a/’ and averaged to obtain the PRP of phoneme ‘ㅏ /a/’.

There are 20 noun-related phonemes and 20 vowel-related phonemes in the Korean storybook, as listed in Table 1. The phoneme onset was extracted from the storybook using Praat software (Paul Boersma and David Weenink, Phonetic Sciences, University of Amsterdam, Netherlands), which is an open-source software program for speech phonetics [16, 17]. The utterance rates of the phonemes are summarized in Table 1. In this study, phonemes with a sufficient number of utterances (n > 100) were used to reliably compute the ensemble neural activity. There was no statistical difference (*p * =  0.782) in the number of phoneme utterances of natural and noise-vocoded speech stories. To compute the PRP of a neural activity in response to a specific phoneme [12], we segmented the EEG signals before/after phoneme onset (0 ms) of the utterance time to have each interval of 100–600 ms, as shown in Fig 2. Fig 2 shows an example of the PRP of a typical phoneme /a/ obtained by averaging 100 segmented EEG signals. As spectral dominance of PRPs was observed in the range of 4–9 Hz, the PRPs were post-processed using a bandpass (2–15 Hz) filter [12].

**Table 1:** Number of utterances for each phoneme in the original and vocoded storybooks. The phonemes with incidences of over 100 were used to compute the phoneme-related potential (PRP). The PRP of ‘ㅢ /ui/’ (marked ‘*’) was particularly computed to compare with the vocoded PRP. The number in parentheses denotes the number of averaging single-trials PRP.

Natural storybook				Vocoded storybook			
Vowel		Consonant		Vowel		Consonant	
Phoneme	Incidence	Phoneme	Incidence	Phoneme	Incidence	Phoneme	Incidence
ㅏ /a/	1494(1453)	ㄴ /n/	1296(1268)	ㅏ /a/	1541(1453)	ㄴ /n/	1471(1268)
ㅣ /i/	812(798)	ㄱ /g/	874(857)	ㅣ /i/	920(798)	ㄱ /g/	931(857)
ㅡ /eu/	736(721)	ㄷ /d/	800(762)	ㅡ /eu/	857(721)	ㄷ /d/	793(762)
ㅓ /eo/	559(548)	ㄹ /l/	595(565)	ㅓ /eo/	679(548)	ㄹ /l/	590(565)
ㅗ /o/	568(493)	ㅁ /m/	486(476)	ㅗ /o/	509(493)	ㅁ /m/	532(476)
ㅜ /u/	326(321)	ㅅ /s/	452(444)	ㅜ /u/	367(321)	ㅅ /s/	530(444)
ㅐ /ae/	279(274)	ㅈ /dz/	407(393)	ㅐ /ae/	311(274)	ㅈ /dz/	413(393)
ㅔ /e/	251(239)	ㄹ /r/	342(339)	ㅔ /e/	250(239)	ㄹ /r/	423(339)
ㅕ /yeo/	244(232)	ㅎ /h/	368(344)	ㅕ /yeo/	242(232)	ㅎ /h/	358(344)
ㅢ /ui/	70(69)*	ㅂ /b/	270(265)	ㅢ /ui/	106(69)	ㅂ /b/	326(265)
		ㅇ /ng/	251(229)			ㅇ /ng/	235(229)
		ㅊ /ch/	87(86)*			ㅊ /ch/	119(86)

Although the frequency of occurrence of each phoneme exceeds 100, the frequencies of occurrence of a phoneme differ between the natural and vocoded stories. The number of averaging single-trials PRP was matched to the smaller of the two, as noted in parentheses in Table 1.

### 2.5. Statistical analysis

The PRPs were calculated by averaging single trials of EEG signals in response to a phonemic stimulus. The amplitude and latency of the PRP component of P1, N1, and P2 were determined and subsequently compared between two experimental conditions (natural and vocoded story) within a subject. As this study was designed with only two conditions, the paired *t*-test was used to investigate if a statistically significant difference existed. Furthermore, the statistically significant difference over time in PRP waveforms between two conditions was determined by computing the *t*-value using a paired *t*-test at each time point.

## 3. Results

### 3.1. Comparison of the grand-averaged PRPs of natural and vocoded speech stories

**Fig 3:**
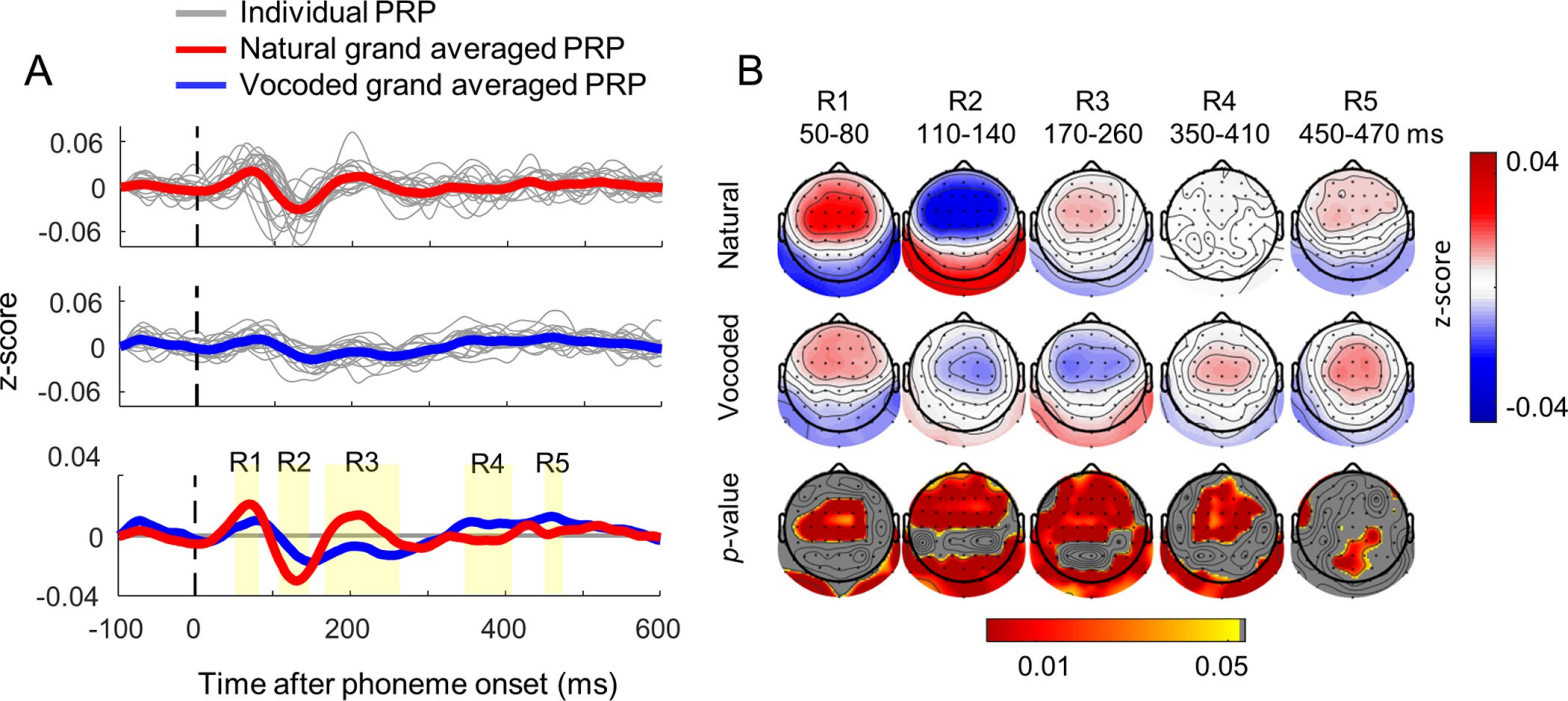
(A) Grand-averaged phoneme-related potential (PRP) computed using the mean PRP of all participants, all phonemes, and all channels. The grand-averaged PRPs in response to natural storybook (red) and vocoded storybook (blue) are plotted in each panel. The PRPs from each subject are plotted in gray. (B) Average topographic map of z-score across participants in the intervals R1 (50–80 ms), R2 (110–140 ms), R3 (170–260 ms), R4 (350–410 ms), and R5 (450–470 ms) in response to natural storybook (first row) and vocoded storybook (second row). The *p*-values of the *t*-test (third row) are colored in red (< 0.01) to yellow (< 0.05).

Fig 3A shows the average PRPs in response to all natural (upper panel) and vocoded phonemes (middle panel). The gray thin line in each panel denotes the averaged PRP across all channels and all phonemes for each participant. The grand-averaged PRPs are indicated by a thick line, and the PRPs from each subject were plotted using a thin gray line. The lower panel in Fig 3A shows a comparison of the grand-averaged PRPs to natural (red) and vocoded (blue) storybooks. Differences on the P1, N1, and P3 components in the grand-averaged PRPs were clearly observed. Higher and earlier peaks in each component in natural PRP are prominent. Here, the *t*-values at each time point over time were computed by a paired *t*-test between natural and vocoded grand averaged PRPs. The intervals of R1 (50–80 ms), R2 (110–140 ms), R3 (170–260 ms), R4 (350–410 ms), and R5 (450–470 ms) were set based on the statistical differences (*p* < 0.05) between two plots as seen in the bottom panel of Fig 3A. To understand the neural processing in the brain at each interval, a topographical PRP map was obtained by averaging the PRPs within each interval at a channel (Fig 3B). The *p*-value topographic map (a paired t-test) shows the statistical differences between two topographic PRPs, as shown in the third row of Fig 3B. Stronger responses in natural PRP than in vocoded PRP are shown in the frontocentral area at early R1 (positive) and R2 (negative), whereas the vocoded PRP was stronger in the late R4 interval.

**Fig 4:**
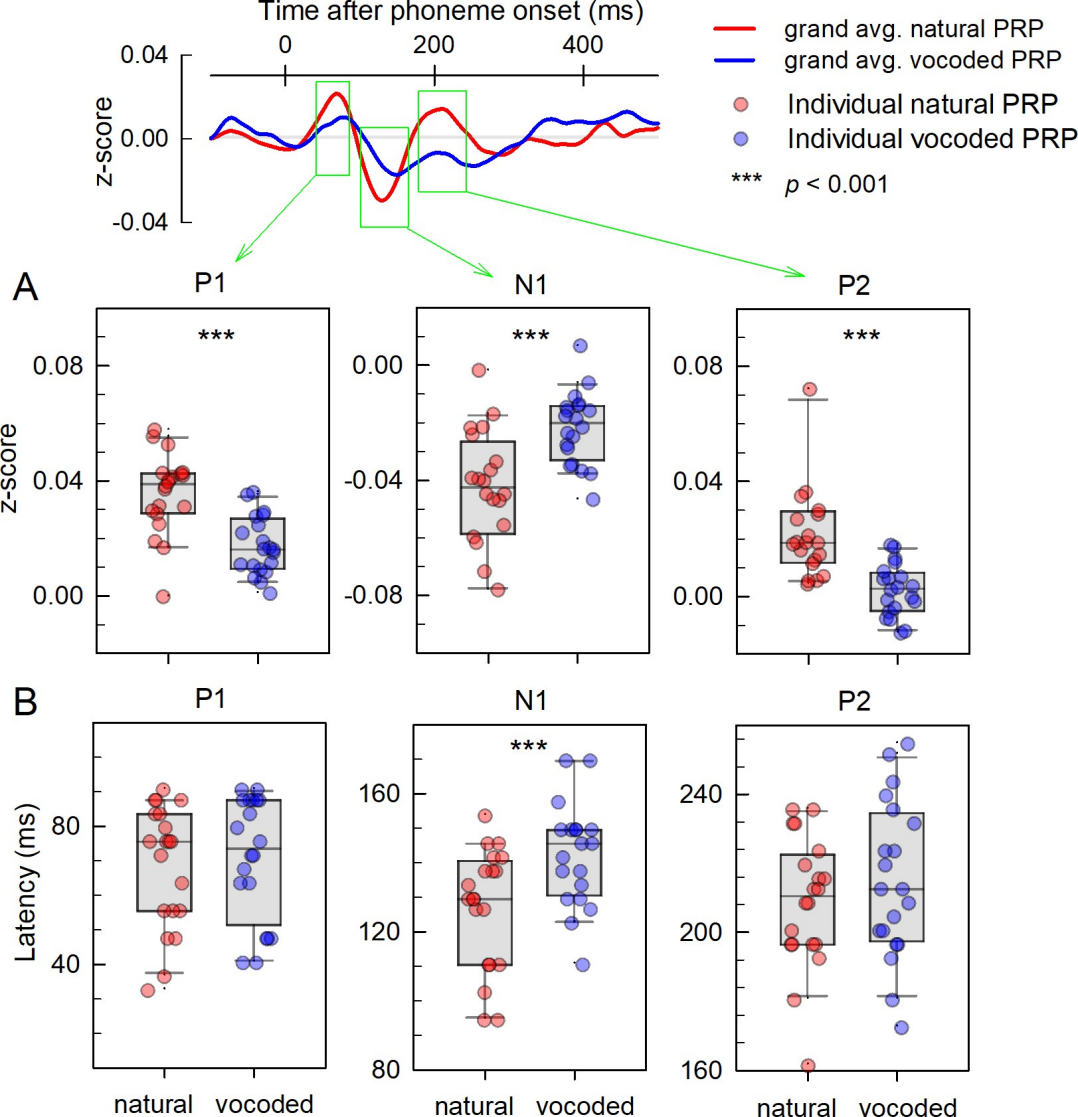
Comparison of the P1-N1-P2 complex of phoneme-related potentials (PRPs) in response to natural and vocoded storybooks. (A) The z-score of the amplitude of each peak P1, N1, and P2 of the PRPs evoked by vocoded and natural storybooks. The gray-circle denotes individual (each subject) data. (B) Peak latency of P1, N1, and P2 of PRP. The latencies were measured after each phoneme onset. The statistical analysis yielded the significant difference (***: *p *< 0.001) between the vocoded and natural cases.

Fig 4 describes the effect of speech degradation on amplitude and latency of the PRP component. In Fig 4, each dot represents the z-score and latency of the grand-averaged PRP across all phoneme stimuli and 64 channels in each subject. A paired *t*-test was conducted to compare natural and vocoded cases. The statistical analysis indicates that the P1, N1, and P2 peaks are significantly larger in natural PRP than in vocoded PRP (***: *p *< 0.001, paired *t*-test) as seen in Fig 4A. Although prolonged mean latencies were observed in the P1, N1, and P2 peaks in vocoded PRP compared with those in natural PRP, a significantly longer latency was only observed in the N1 peak, as shown in the lower panel in Fig 4B (***: *p *< 0.001, paired *t*-test) and Table 2.

**Table 2:** Summary of PRP peak amplitude, latency, and statistical comparison between natural and vocoded cases.

z-score (amplitude)		Mean	SD	*p*-value
P1	natural	0.035	0.014	*p* < 0.001
	vocoded	0.016	0.011	
N1	natural	-0.022	0.010	*p* < 0.001
	vocoded	-0.043	0.017	
P2	natural	0.022	0.016	*p* < 0.001
	vocoded	0.002	0.009	
Latency (ms)		Mean	SD	*p*-value
P1	natural	68.6	17.7	-
	vocoded	71.6	17.9	
N1	natural	127.0	17.0	*p* < 0.001
	vocoded	146.9	18.8	
P2	natural	209.2	18.9	-
	vocoded	211.1	23.5	

SD: standard deviation; *p*-value: between the paired (natural and vocoded cases in a subject) samples

### 3.2. Comparison of individual phoneme-related potentials

**Fig 5:**
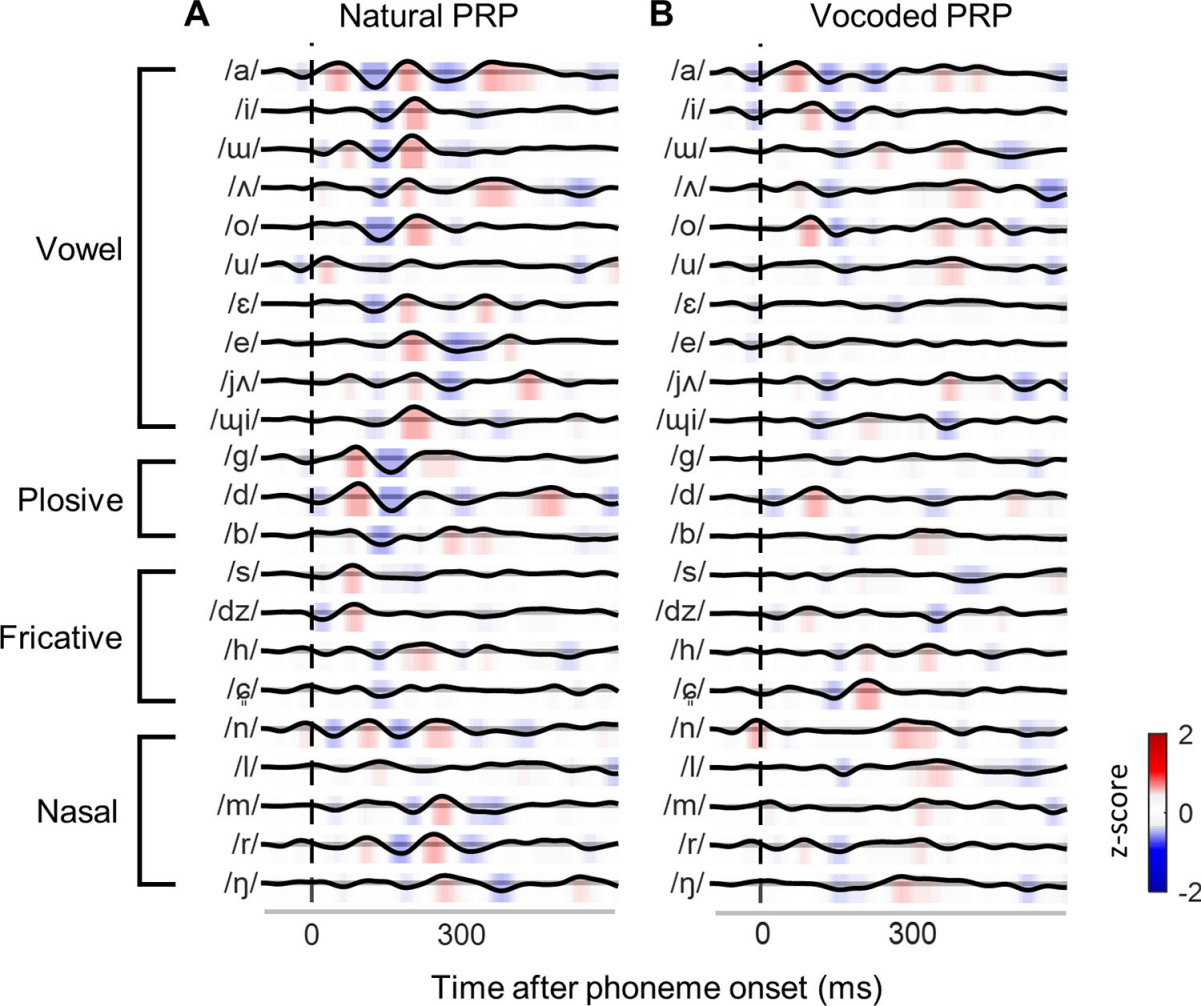
Comparison of phoneme-related potential (PRP) waveforms in response to natural and vocoded storybooks. The PRPs were plotted in articulate (vowel, plosive, fricative, and nasal) and alphabetical order. The color in each PRP indicates positive (red) and negative (blue) z-scores.

Fig 5 shows the natural and vocoded PRPs for each 10 vowels and 12 consonants. Each PRP at FCz was averaged across the participants. The color bar at each PRP component provides intuitive z-score information about the positive (red) and negative (blue) amplitudes. The manner of articulation clearly affects the amplitude and latency of the PRP waveform. There is a significant decrease in the amplitude of vocoded PRP compared with that of natural PRP.

**Fig 6:**
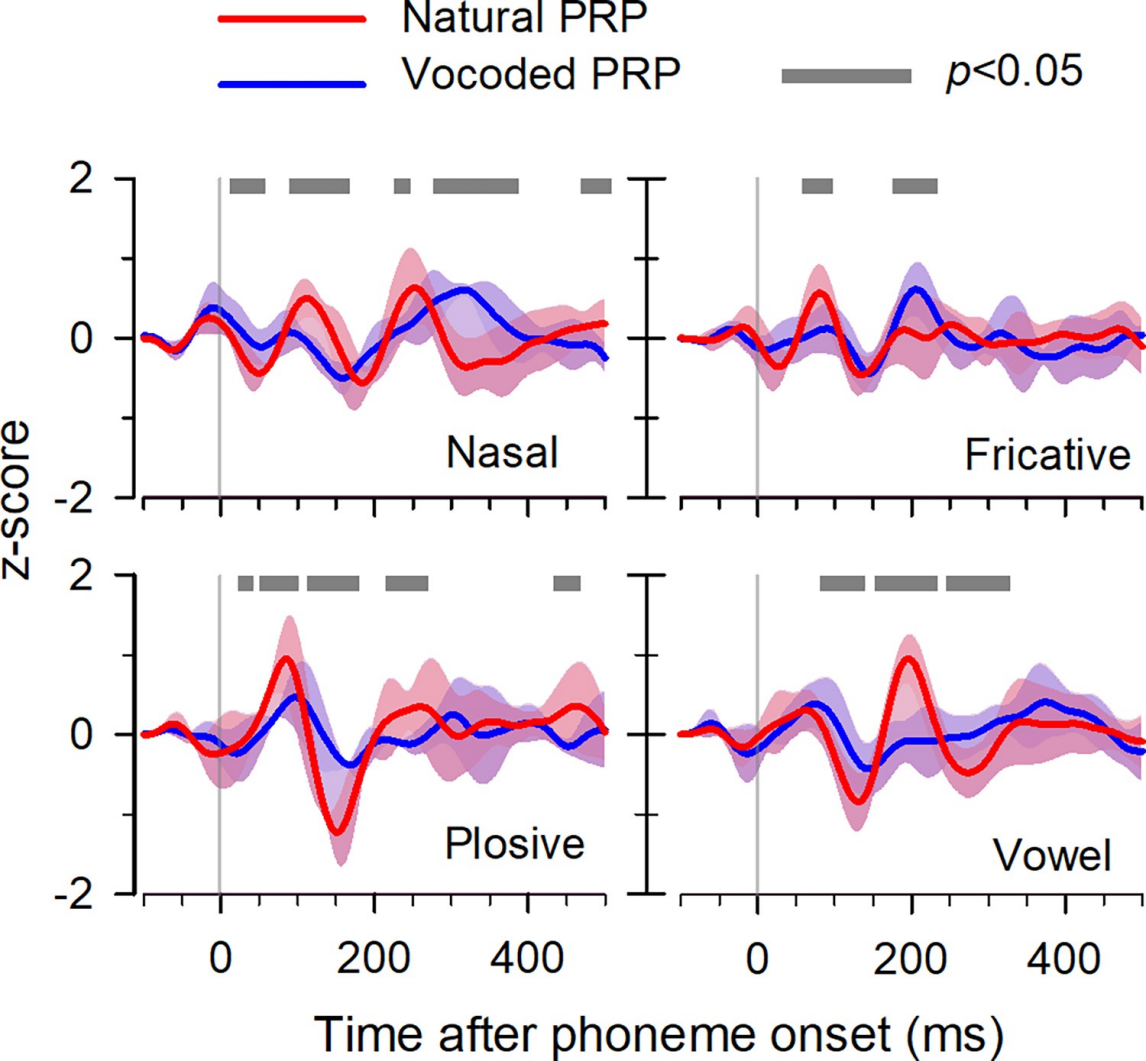
Grand-averaged phoneme-related potentials in an articulate group (nasal, fricative, plosive, and vowel) in response to natural (red) and vocoded (blue) storybooks. The transparent area indicates the range of individual data. The gray bars denote the statistically (*p *< 0.01, *t*-test) different intervals.

The comparisons of the natural and vocoded PRPs in response to vowels and plosive-, fricative-, and nasal-consonants are shown in Fig 6. Each panel shows plots of the averaged PRPs at FCz across each phoneme group of 10 vowels, 3 plosives, 4 fricatives, and 5 nasals. Although the incidence of each phoneme varied, as shown in Table 1, the averaged PRP for each phoneme stimulation could be calculated sufficiently. The averaged PRPs from the subjects were employed to investigate the statistical differences depending on manner and place of articulation. The gray-bar in each panel represents the statistical difference over time between the averaged PRPs of the natural and vocoded cases (*p *< 0.01, paired *t*-test). It was noted that significant differences were observed in the R1 (50–80 ms), R2 (110–140 ms), R3 (170–260 ms), and R4 (350–410 ms) intervals of the grand-averaged PRP following both natural and vocoded storybook stimulation, as illustrated in Fig 3. The results were consistent across all four cases examined in this section, categorized by phonetic groups based on articulation. Significant differences were found between natural and vocoded PRPs in peak amplitudes during early R1 (50–80 ms) and R2 (110–140 ms) intervals, with higher amplitudes in natural PRPs. Conversely, during late R3 (170–260 ms) and R4 (350–410 ms) intervals, higher amplitudes were observed in vocoded PRPs for nasal, plosive, and vowel sounds. The late latency component of the vocoded PRPs was observed for the phonemes of vowels, nasals, and plosives.

## 4. Discussion

In this study, we evaluated the effect of speech degradation on PRP in response to a continuous storybook. The latency and amplitude of PRP in natural continuous storybooks were significantly shorter and higher, respectively, than those in vocoded storybooks.

### 4.1. Similarity to late AEP P1-N1-P2

The latter AEPs are generally known to be involved in stimulus recognition and information processing. PRP has the latter components of P1-N1-P2 that are similar to those of general AEP that occur during central auditory processing (see Fig 4). P1 (first positive peak) of PRP was observed between 40 ms and 75 ms, N1 (first negative peak) between 90 ms and 200 ms, and P2 (second positive peak) between 100 ms and 250 ms after stimulus onset. It has been reported that the amplitude and latency of P1-N1-P2 are useful to objectively evaluate auditory functions [18, 19]. The current study revealed that the amplitude and latency of the PRP peak varied depending on speech intelligibility, which is consistent with the findings of a previous AEP study [20].

### 4.2. Phoneme related potential according to phoneme class

Phonemes are generally classified according to the place of articulation during their utterances [21]. Kovács et al. (2017) reported that different AEPs occurred in response to syllabic nonsense words based on the phoneme classes (fricative, plosive, nasal, affricate, and liquid) [22]. It has been demonstrated that the human auditory system is effectively sensitive to sudden changes in spectrotemporal information [23]. The results of this study highlight the consistency in the effect of speech-stimulus degradation in each phoneme class on PRP, as seen in Figs 5 and 6. However, the similarity of PRP waveforms within each phoneme class was not observed. It is necessary to evaluate the similarity of waveforms classified based on stimulus feature such as fundamental frequency rather than utterances.

### 4.3. Clinical relevance for the prediction of speech intelligibility

Behavioral speech tests have been used to evaluate speech recognition in clinical settings. An objective approach using continuous speech-evoked EEG has also been recently proposed [3, 24]. The repetitive presentation of words or short sentences to compute the synchronized AEP deteriorates the task performance efficiency [6, 25]. Because running speech stimuli with a story, which is not repetitive, can arouse the subject’s interest in maintaining attention, PRP has the advantage of evaluating speech intelligibility compared with conventional AEP tests as well as understanding phonemic awareness.

### 4.4. Limitations on phoneme related potential

The phonemic awareness, which represents the ability of separating and identifying individual phonemes in spoken words, can be assessed by behavioral tasks or objective ERPs [26, 27] This study showed that PRP can be a valuable tool to test the ability of hearing individual sounds in words. Coarticulation sensitivity, the ability to perceive the overlap phonemes, also helps individuals to better understand spoken language, even in challenging listening conditions. Several studies have used the mismatch negative component of ERP to allow the processing of vowel-consonant or vowel-vowel coarticulation [28, 29]. While the use of PRP has provided insights into the phonemic processing, limitations exist in our understanding of the underlying coarticulation. Therefore, further research is needed to fully explore and comprehend the cognitive and neural processes involved in perceiving individual phonemes.

## 5. Conclusions

This study demonstrated that significant differences occurred in the grand-averaged PRP as well as the PRP in response to each phoneme between the natural and vocoded cases. These findings indicate that PRP can be used as an objective measure to evaluate speech intelligibility in clinical settings. However, some issues need to be addressed prior to clinical implementation. The EEG data were acquired from only normal hearing subjects using natural and vocoded story stimuli. Therefore, additional validations for subjects with hearing impairment should be performed. Moreover, as the phonemes in the storybook used in this study were asymmetrically distributed, typical phonemes were limited to achieve reliable PRPs with a high signal-to-noise ratio. A storybook that thoroughly covers a sufficient number of phonemes should be further examined.
